# Wernicke’s encephalopathy in a patient with adenoid cystic carcinoma: case report and literature review

**DOI:** 10.3389/fonc.2025.1723422

**Published:** 2025-11-26

**Authors:** Ioannis Kotsantis, Anna Boulouta, Panagiota Economopoulou, Anastasios Kyriazoglou, Sotirios Karamagkiolas, Maria Kyrkasiadou, Niki Gavrielatou, Maria Anastasiou, Anastasios Pantazopoulos, Amanda Psyrri

**Affiliations:** 1 Second Department of Internal Medicine, Oncology Unit, Attikon University Hospital, National and Kapodistrian University of Athens, School of Medicine, Athens, Greece; 2 Second Otolaryngology Department, Attikon University Hospital, National and Kapodistrian University of Athens, School of Medicine, Athens, Greece

**Keywords:** Wernicke’s encephalopathy, head & neck cancer, adenoid cystic carcinoma, nutritional complications, thiamine deficiency, neurological manifestation, HNSCC

## Abstract

Wernicke’s encephalopathy is an acute neurologic disorder caused by thiamine (vitamin B1) deficiency, which is most commonly associated with alcoholism. Rare cases of Wernicke’s encephalopathy have been described in cancer patients, mostly with gastrointestinal and hematologic malignancies. Head and neck cancer patients frequently have reduced oral intake as a direct result of their tumor or from chemoradiation treatments. We report a case of Wernicke’s encephalopathy in a 44-year-old woman with adenoid cystic carcinoma following chemoradiotherapy. Through a literature review we identified additional cases of Wernicke’s encephalopathy in head and neck cancer patients, highlighting the importance of recognizing nutritional deficiencies and associated complications. The findings emphasize the need for heightened awareness regarding the risk of thiamine deficiency in cancer patients, particularly those experiencing poor nutritional intake due to treatment-related side effects. Prompt diagnosis and intervention are critical to prevent serious morbidity and mortality associated with this condition.

## 1 Introduction

Head and neck cancers are a heterogeneous group of malignant tumors that include squamous cell carcinoma of the head and neck, nasopharyngeal carcinoma and salivary gland tumors (1). Locoregional disease is treated with curative intent surgery or chemoradiation (CRT), with the most frequently used chemotherapy regimens using cisplatin, carboplatin, 5-fluorouracil (5-FU) and docetaxel (2).

The most common adverse events of CRT are mucositis, xerostomia, dysphagia, trismus, dermatitis, fatigue, nausea, vomiting and loss of taste (3, 4). Patients may be malnourished at presentation, and this may worsen during the course of treatment depending on tumor location and treatment tolerance.

Neurotoxicity is a known and potential dose-limiting complication of CRT for head and neck cancer. Platinum agents and taxanes can cause peripheral neuropathy, while 5-FU can rarely cause encephalopathy or cerebellar syndrome (5–7). Radiotherapy may also induce neurologic toxicity due to the close proximity of tumor targets to critical neurologic structures, including the brain and cranial nerves. As a result, neurologic sequelae such as radiation-induced neuropathy and brainstem toxicity may arise, even though these usually present as a late event (8).

Wernicke’s encephalopathy is an acute neurologic disorder caused by thiamine (vitamin B1) deficiency, which disrupts normal brain function. It is characterized by a triad of symptoms including confusion, ataxia and ophthalmoplegia. While Wernicke’s encephalopathy is commonly linked to alcoholism, it can also occur in non-alcoholics due to various factors such as malnutrition, gastrointestinal disorders and systemic disorders. Severe malnutrition directly results in inadequate thiamine intake due to poor dietary habits. Additionally, conditions such as infections and other systemic disorders can elevate the basal metabolic rate, leading to an increased demand for thiamine and subsequently inducing deficiency (9, 10).

Wernicke’s encephalopathy has been described in cancer patients, especially in patients with gastrointestinal malignancies, due to poor absorption and surgical resection of parts of the gastrointestinal tract (11–17), as well as in hematologic malignancies (18–21). Additionally, in rare cases it has also been linked to the use of 5-FU, which induces increased thiamine metabolism (22–24).

## 2 Case presentation

We present a case of a 44-year-old woman with a history of adenoid cystic carcinoma of the nasal cavity diagnosed four months prior to admission, stage IVB (TNM T4N0M0 according to AJCC cancer staging eighth edition [2017]) for salivary gland tumors. She underwent surgical removal of the tumor with positive margins, followed by adjuvant chemoradiotherapy (three cycles of cisplatin 100mg/m^2^ q21d, and radiotherapy with a total dose of 60Gy). She had completed concurrent CRT treatment two months before her current presentation. During the last two cycles of treatment, she developed hyperemesis and dysphagia due to treatment toxicity, which limited her oral intake. Her past medical history was otherwise unremarkable.

The patient presented to the emergency department with a 10-day history of mental status changes, disorientation, behavioral changes, ataxia and paraplegia. On physical examination she was noted to have ophthalmoplegia, horizontal nystagmus, bilateral ptosis, as well as complete inability to stand. She was confused and stuporous. Neurological examination showed no meningeal signs. Blood tests, including complete blood count, basic metabolic panel, liver function tests and inflammatory markers were normal. Brain CT scan showed no abnormalities and no evidence of residual or recurrent tumor. The patient was admitted to the hospital for work-up.

**Figure 1:**
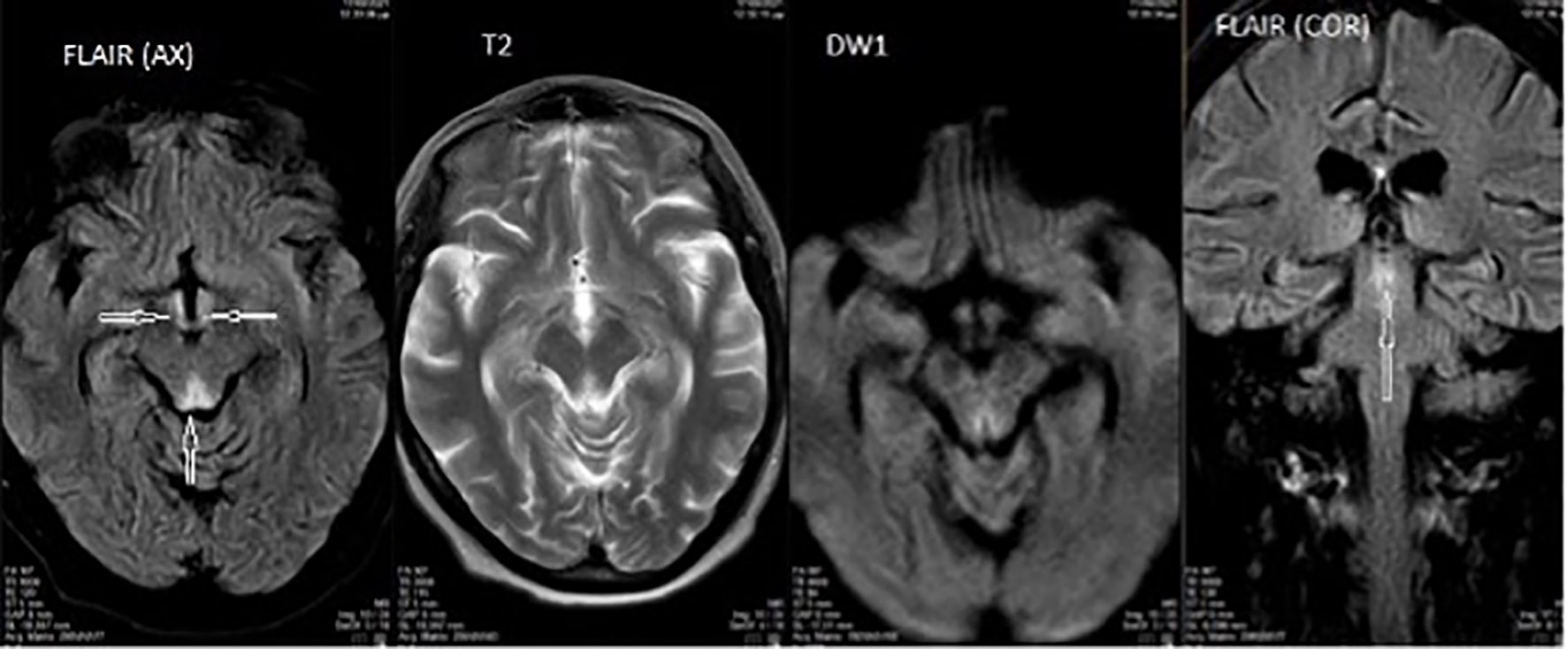
Axial and coronal brain MRI demonstrating T2/FLAIR hyperintensity in the periaqueductal gray matter of the upper pons and midbrain, consistent with findings typically seen in Wernicke’s encephalopathy.

Five days after admission the patient remained stuporous, with further deterioration of her mental status and totally unable to perform any activity. Brain MRI was performed and T2 images revealed abnormal signal in the periaqueductal gray matter of the upper pons and midbrain area (**Figure 1**), findings that are typical for Wernicke’s encephalopathy.

**Figure 2:**
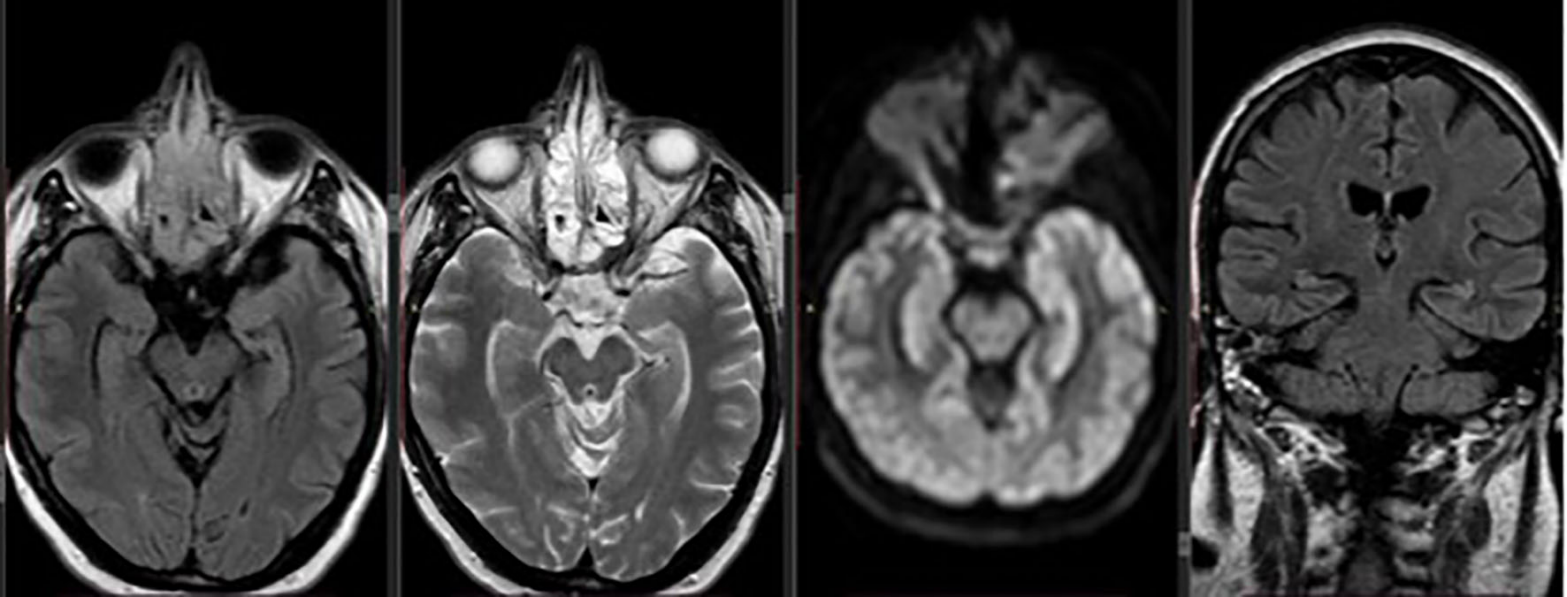
Axial and coronal follow-up brain MRI showing complete resolution of the previously noted periaqueductal hyperintensities, with no remaining signs of encephalopathy.

Based on clinical deterioration and imaging findings, thiamine supplementation was started immediately at a dose of 500 mg i.v three times daily for two days and 250 mg once daily for five days, with the patient showing rapid improvement in her mental status and ophthalmoplegia. Two days following treatment initiation, she had resumed memory and communication, she had no nystagmus and no vision problems. However, she remained unable to stand and walk. For that reason, physiotherapy was started allowing the patient gradual improvement over the following days, and she was discharged from the hospital with oral thiamine supplementation to maintain adequate levels and prevent recurrence. Three months later, during the follow-up visit, she remained paraplegic, without any other neurological symptoms and her follow-up MRI showed no evidence of encephalopathy (**Figure 2**). The patient died two years later due to disease progression.

## 3 Literature review

A literature review was conducted in PubMed. The terms “Wernicke’s”, “encephalopathy”, “head and neck cancer”, “oropharyngeal”, “rhinopharyngeal”, “salivary gland” and all combinations of those terms were used in our literature search. The search yielded 7 relevant case reports and case series presenting a total of 8 cases, of which 5 were patients head and neck squamous cell carcinomas (HNSCC) and 3 were nasopharyngeal carcinomas (NPC). The majority of cases had received CRT, while one had only received radiation therapy (RT). All cases are listed in **Table 1**.

**Table 1:** Reported cases of Wernicke’s encephalopathy in patients with head and neck malignancies.

No	Author, year	Malignancy	Treatment modality
1	Fikhman, 2011 (25)	HNSCC of the tongue	Surgery + CRT
2	Fikhman, 2011 (25)	HNSCC of the tonsil	CRT
3	Cho, 2009 (22)	NPC	ChT (CDDP) +RT, CDDP/5FU
4	Zeng, 2015 (26)	HNSCC	ChT (Carboplatin/5FU) + RT
5	Law, 2011 (27)	NPC	ChT (CDDP) + RT
6	Kosta, 2005 (28)	NPC	ChT (docetaxel-based) + RT
7	Choi, 2016 (29)	HNSCC of the larynx	ChT (TPF) + RT + surgery
8	Isenberg-Grzeda, 2015 (30)	HNSCC of the eyebrow	RT

HNSCC, head and neck squamous cell carcinoma; CRT, chemoradiotherapy; NPC, nasopharyngeal carcinoma; ChT, chemotherapy; CDDP, cisplatin; 5FU, 5-fluorouracil; TPF, docetaxel, cisplatin, fluorouracil.

## 4 Discussion

There is emerging research that suggests association between cancer and the development of Wernicke’s encephalopathy. Patients with a malignant tumor are highly susceptible to this acute encephalopathy because of prolonged malnutrition, nausea and vomiting caused by chemotherapy, as well as the depletion of thiamine by fast-growing tumors and increased basal metabolic rate. Most case reports highlight substantial barriers to sufficient nutritional intake in cancer patients, including persistent nausea and vomiting, loss of appetite, mucositis, obstruction, and reliance on total parenteral nutrition. Additionally, several case reports document patients with malignancies who lack any identifiable nutritional barriers or other known risk factors for Wernicke’s encephalopathy, indicating that malignancies themselves may serve as an independent risk factor for developing this condition (29–31).

Patients with head and neck cancer are highly susceptible to inadequate nutrition due to mechanical obstruction, pain, and trismus. These factors, combined with CRT toxicity such as nausea, vomiting, stomatitis and dysphagia, lead to poor nutritional intake.

Recent evidence has highlighted additional mechanisms contributing to thiamine deficiency in cancer patients with impaired enteral intake. Beyond decreased oral intake, thiamine depletion may arise from poor intestinal absorption, surgical resection of gastrointestinal segments, prolonged reliance on parenteral nutrition without adequate supplementation, and increased metabolic demand driven by tumor proliferation. These factors may interact to accelerate thiamine depletion and precipitate Wernicke’s encephalopathy even in the absence of classic nutritional risk profiles (32).

Many critically ill cancer patients may experience altered mental status due to a variety of overlapping factors, such as hypoxia, infections, electrolyte imbalances, medications and central nervous system metastases. While Wernicke’s encephalopathy is a rare entity, clinicians should remain especially vigilant and maintain a high suspicion for thiamine deficiency when nutritional intake is severely compromised. High clinical suspicion should prompt imaging with MRI and rapid initiation of treatment in order to prevent irreversible brain damage and possible fatal consequences. With growing research on the prevalence of thiamine deficiency and Wernicke’s encephalopathy in cancer patients, providers are likely to recognize this entity more frequently. It’s essential for healthcare professionals to recognize the clinical presentation and causes of this neurological emergency to accurately diagnose and treat it effectively, reducing the associated risks of serious morbidity and mortality.

In patients with poor oral intake or high risk of thiamine deficiency, early supplementation is recommended before neurological symptoms develop. For patients at risk of thiamine deficiency due to poor nutritional intake, prophylactic supplementation is generally recommended at approximately 250 mg of thiamine administered intravenously (IV) or intramuscularly (IM) once daily for 3 to 5 days (9, 33). In confirmed or strongly suspected Wernicke’s encephalopathy, higher parenteral dosing is advised. Guidelines from A.S.P.E.N. and other expert sources support using 500 mg IV three times daily for the first 2 to 3 days, followed by 250 mg IV or IM once daily for an additional 3 to 5 days (9, 33). Notably, high-dose regimens up to 1000 mg IV daily have been used in clinical practice without reported adverse effects (34). Maintenance oral thiamine is recommended after the initial high IV doses in non-alcoholic Wernicke encephalopathy at a dose of 50–100 mg/day, especially if nutritional risk persists (9, 10). Because thiamine deficiency often coexists with other micronutrient deficiencies, concurrent administration of other B vitamins and multivitamin preparations is recommended (33, 35).

## 5 Conclusions

Thiamine deficiency and Wernicke’s encephalopathy have rarely been described in patients with head and neck cancer. Due to impaired dietary intake as a result of CRT side effects, our patient developed Wernicke’s encephalopathy. Following prompt workup and imaging, thiamine supplementation was administered, and the patient had a partial reversal of symptoms. In cancer patients presenting with altered mental status, ataxia or ophthalmoplegia, thiamine deficiency should always be considered and, if confirmed, treated urgently.

## Data Availability

The original contributions presented in the study are included in the article/supplementary material. Further inquiries can be directed to the corresponding author.

## References

[1] Gormley M Creaney G Schache A Ingarfield K Conway DI . Reviewing the epidemiology of head and neck cancer: definitions, trends and risk factors. Br Dent J. (2022) 233:780–6. doi: 10.1038/s41415-022-5166-x, PMID: 36369568 PMC9652141

[2] Machiels JP René Leemans C Golusinski W Grau C Licitra L Gregoire V . Electronic address: secretariat@ehns.org; ESMO Guidelines Committee. Electronic address: clinicalguidelines@esmo.org; ESTRO Executive Board. Electronic address: info@estro.org. Squamous cell carcinoma of the oral cavity, larynx, oropharynx and hypopharynx: EHNS-ESMO-ESTRO Clinical Practice Guidelines for diagnosis, treatment and follow-up. Ann Oncol. (2020) 31:1462–75. doi: 10.1016/j.annonc.2020.07.011, PMID: 33239190

[3] Tang LL Guo R Zhang N Deng B Chen L Cheng ZB . Effect of radiotherapy alone vs radiotherapy with concurrent chemoradiotherapy on survival without disease relapse in patients with low-risk nasopharyngeal carcinoma: A randomized clinical trial. JAMA. (2022) 328:728–36. doi: 10.1001/jama.2022.13997, PMID: 35997729 PMC9399866

[4] Posner MR Hershock DM Blajman CR Mickiewicz E Winquist E Gorbounova V . Cisplatin and fluorouracil alone or with docetaxel in head and neck cancer. N Engl J Med. (2007) 357:1705–15. doi: 10.1056/NEJMoa070956, PMID: 17960013

[5] Macdonald DR . Neurologic complications of chemotherapy. Neurol Clin. (1991) 9:955–67. doi: 10.1016/S0733-8619(18)30259-7 1758434

[6] Sul JK Deangelis LM . Neurologic complications of cancer chemotherapy. Semin Oncol. (2006) 33:324–32. doi: 10.1053/j.seminoncol.2006.03.006, PMID: 16769421

[7] Boilève A Thomas L Lillo-Le Louët A Gaboriau L Chouchana L Ducreux M . 5-Fluorouracil-induced hyperammonaemic encephalopathy: A French national survey. Eur J Cancer. (2020) 129:32–40. doi: 10.1016/j.ejca.2020.01.019, PMID: 32120273

[8] Chow JCH Ho JCS Cheung KM Johnson D Ip BYM Beitler JJ . Neurological complications of modern radiotherapy for head and neck cancer. Radiother Oncol. (2024) 194:110200. doi: 10.1016/j.radonc.2024.110200, PMID: 38438018

[9] Sechi G Serra A . Wernicke’s encephalopathy: new clinical settings and recent advances in diagnosis and management. Lancet Neurol. (2007) 6:442–55. doi: 10.1016/S1474-4422(07)70104-7, PMID: 17434099

[10] Sinha S Kataria A Kolla BP Thusius N Loukianova LL . Wernicke encephalopathy-clinical pearls. Mayo Clin Proc. (2019) 94:1065–72. doi: 10.1016/j.mayocp.2019.02.018, PMID: 31171116

[11] Jung ES Kwon O Lee SH Lee KB Kim JH Yoon SH . Wernicke’s encephalopathy in advanced gastric cancer. Cancer Res Treat. (2010) 42:77–81. doi: 10.4143/crt.2010.42.2.77, PMID: 20622961 PMC2901082

[12] Papila B Yildiz O Tural D Delil S Hasiloglu ZI Ayan F . Wernicke’s encephalopathy in colon cancer. Case Rep Oncol. (2010) 3:362–7. doi: 10.1159/000321457, PMID: 21537379 PMC3085069

[13] Rakici SY Erdemli SD Yazici ZA Cengiz E Acar OG Tufan G . Wernicke’s encephalopathy in a patient with unresectable gastric carcinoma and literature review. Int J Clin Exp Med. (2015) 8:1453–9., PMID: 25785154 PMC4358609

[14] Restivo A Carta MG Farci AMG Saiu L Gessa GL Agabio R . Risk of thiamine deficiency and Wernicke’s encephalopathy after gastrointestinal surgery for cancer. Support Care Cancer. (2016) 24:77–82. doi: 10.1007/s00520-015-2748-z, PMID: 25931232

[15] Nikjoo A Rashid H Chung R Sadat MA . A rare case of Wernicke encephalopathy in stage IV gastric cancer. Neurocase. (2022) 28:123–5. doi: 10.1080/13554794.2022.2041043, PMID: 35188084

[16] Zhang Y Wang L Jiang J Chen WY . Non-alcoholic Wernicke encephalopathy in an esophageal cancer patient receiving radiotherapy: A case report. World J Clin Cases. (2022) 10:5810–5. doi: 10.12998/wjcc.v10.i17.5810, PMID: 35979132 PMC9258394

[17] Li Q Wang F Cheng L Chen L Wu Z . Wernicke encephalopathy following advanced caecum cancer. Rev Esp Enferm Dig. (2021) 113:856–7. doi: 10.17235/reed.2021.8343/2021, PMID: 34538057

[18] Shah N Wolff JA . Thiamine deficiency: probable Wernicke’s encephalopathy successfully treated in a child with acute lymphocytic leukemia. Pediatrics. (1973) 51:750–1. doi: 10.1542/peds.51.4.750, PMID: 4512248

[19] Brück W Christen HJ Lakomek H Hanefeld F Friede RL . Wernicke’s encephalopathy in a child with acute lymphoblastic leukemia treated with polychemotherapy. Clin Neuropathol. (1991) 10:134–6., PMID: 1860271

[20] Nakajima D Fukushima K Yamanouchi H . Neurological complications during and after the treatment of acute lymphocytic leukemia. No To Hattatsu. (2006) 38:195–200. doi: 10.1055/s-2006-945726, PMID: 16715933

[21] Boniol S Boyd M Koreth R Burton GV . Wernicke encephalopathy complicating lymphoma therapy: case report and literature review. South Med J. (2007) 100:717–9. doi: 10.1097/SMJ.0b013e318061920a, PMID: 17639753

[22] Cho IJ Chang HJ Lee KE Won HS Choi MY Nam EM . A case of Wernicke’s encephalopathy following fluorouracil-based chemotherapy. J Korean Med Sci. (2009) 24:747–50. doi: 10.3346/jkms.2009.24.4.747, PMID: 19654964 PMC2719188

[23] Heier MS Dornish JM . Effect of the fluoropyrimidines 5-fluorouracil and doxifluridine on cellular uptake of thiamin. Anticancer Res. (1989) 9:1073–7., PMID: 2530931

[24] Kondo K Fujiwara M Murase M Kodera Y Akiyama S Ito K . Severe acute metabolic acidosis and Wernicke’s encephalopathy following chemotherapy with 5-fluorouracil and cisplatin: case report and review of the literature. Jpn J Clin Oncol. (1996) 26:234–6. doi: 10.1093/oxfordjournals.jjco.a023220, PMID: 8765181

[25] Fikhman G Berger JR Gal TJ . Wernicke’s encephalopathy in the course of chemoradiotherapy for head and neck cancer. Am J Otolaryngol. (2011) 32:250–2. doi: 10.1016/j.amjoto.2010.01.009, PMID: 20434810

[26] Zeng KL Kuruvilla S Sanatani M Louie AV . Bilateral blindness following chemoradiation for locally advanced oropharyngeal carcinoma. Cureus. (2015) 7:e352. doi: 10.7759/cureus.352, PMID: 26623207 PMC4652920

[27] Law HL Tan S Sedi R . Wernicke’s encephalopathy in a patient with nasopharyngeal carcinoma: magnetic resonance imaging findings. Malays J Med Sci. (2011) 18:71–4., PMID: 22135604 PMC3216226

[28] Kosta P Margariti P Tolis C Tsimichodimos V Konitsiotis S Argyropoulou M . Wernicke’s encephalopathy in a patient with rhinopharyngeal carcinoma. J Neurol. (2005) 252:1539–40. doi: 10.1007/s00415-005-0877-x, PMID: 16021361

[29] Choi EY Gomes WA Haigentz M Jr Graber JJ . Association between Malignancy and non-alcoholic Wernicke’s encephalopathy: a case report and literature review. Neurooncol Pract. (2016) 3:196–207. doi: 10.1093/nop/npv036, PMID: 31386087 PMC6668278

[30] Isenberg-Grzeda E Hsu AJ Hatzoglou V Nelso C Breitbart W . Palliative treatment of thiamine-related encephalopathy (Wernicke’s encephalopathy) in cancer: A case series and review of the literature. Palliat Support Care. (2015) 13:1241–9. doi: 10.1017/S1478951514001163, PMID: 25339378 PMC4982657

[31] Kuo SH Debnam JM Fuller GN de Groot J . Wernicke’s encephalopathy: an underrecognized and reversible cause of confusional state in cancer patients. Oncology. (2009) 76:10–8. doi: 10.1159/000174951, PMID: 19018150

[32] Koca O Demir B Derin S Turna ZH . A case report of Wernicke Korsakoff syndrome in a patient with cholangiocellular carcinoma: An underestimated cause of encephalopathy in cancer patients. Med (Baltimore). (2022) 101:e31904. doi: 10.1097/MD.0000000000031904, PMID: 36482648 PMC9726370

[33] Vanek VW Borum P Buchman A Fessler TA Howard L Jeejeebhoy K . A.S.P.E.N. position paper: recommendations for changes in commercially available parenteral multivitamin and multi-trace element products. Nutr Clin Pract. (2012) 27:440–91. doi: 10.1177/0884533612446706, PMID: 22730042

[34] Mifsud F Messager D Jannot AS Védie B Balanant NA Poghosyan T . Clinical diagnosis, outcomes and treatment of thiamine deficiency in a tertiary hospital. Clin Nutr. (2022) 41:33–9. doi: 10.1016/j.clnu.2021.10.021, PMID: 34864453

[35] Dingwall KM Delima JF Binks P Batey R Bowden SC . What is the optimum thiamine dose to treat or prevent Wernicke’s encephalopathy or Wernicke-Korsakoff syndrome? Results of a randomized controlled trial. Alcohol Clin Exp Res. (2022) 46:1133–47. doi: 10.1111/acer.14843, PMID: 35428992 PMC9321884

